# A Single Amino Acid Residue Regulates PTEN-Binding and Stability of the Spinal Muscular Atrophy Protein SMN

**DOI:** 10.3390/cells9112405

**Published:** 2020-11-03

**Authors:** Sebastian Rademacher, Nora T. Detering, Tobias Schüning, Robert Lindner, Pamela Santonicola, Inga-Maria Wefel, Janina Dehus, Lisa M. Walter, Hella Brinkmann, Agathe Niewienda, Katharina Janek, Miguel A. Varela, Melissa Bowerman, Elia Di Schiavi, Peter Claus

**Affiliations:** 1 Institute of Neuroanatomy and Cell Biology, Hannover Medical School, 30625 Hannover, Germany; sebastian.rademacher@charite.de (S.R.); Detering.Nora@mh-hannover.de (N.T.D.); Schuening.Tobias@mh-hannover.de (T.S.); Lindner.Robert@mh-hannover.de (R.L.); i-m.wefel@web.de (I.-M.W.); Janina.Dehus@web.de (J.D.); l.walter-enzen@t-online.de (L.M.W.); Brinkmann.Hella@mh-hannover.de (H.B.); 2 Center for Systems Neuroscience (ZSN), 30559 Hannover, Germany; 3 Institute of Biosciences and Bioresources, National Research Council of Italy, 80131 Naples, Italy; pamela.santonicola@ibbr.cnr.it (P.S.); elia.dischiavi@ibbr.cnr.it (E.D.S.); 4 Shared Facility for Mass Spectrometry, Institute of Biochemistry, Charité—Universitätsmedizin Berlin, Corporate Member of Freie Universität Berlin, Humboldt-Universität zu Berlin, and Berlin Institute of Health, 10117 Berlin, Germany; agathe.niewienda@charite.de (A.N.); katharina.janek@charite.de (K.J.); 5 Department of Physiology, Anatomy and Genetics, University of Oxford, Oxford OX1 3QX, UK; miguel.varela@dpag.ox.ac.uk (M.A.V.); m.bowerman@keele.ac.uk (M.B.); 6 Department of Paediatrics, John Radcliffe Hospital, University of Oxford, Oxford OX3 9DU, UK; 7 School of Medicine, Keele University, Staffordshire ST5 5BG, UK; 8 Wolfson Centre for Inherited Neuromuscular Disease, RJAH Orthopaedic Hospital, Oswestry SY10 7AG, UK

**Keywords:** mass spectrometry, phosphorylation, PTEN, SMN, spinal muscular atrophy

## Abstract

Spinal Muscular Atrophy (SMA) is a neuromuscular disease caused by decreased levels of the survival of motoneuron (SMN) protein. Post-translational mechanisms for regulation of its stability are still elusive. Thus, we aimed to identify regulatory phosphorylation sites that modulate function and stability. Our results show that SMN residues S290 and S292 are phosphorylated, of which SMN pS290 has a detrimental effect on protein stability and nuclear localization. Furthermore, we propose that phosphatase and tensin homolog (PTEN), a novel phosphatase for SMN, counteracts this effect. In light of recent advancements in SMA therapies, a significant need for additional approaches has become apparent. Our study demonstrates S290 as a novel molecular target site to increase the stability of SMN. Characterization of relevant kinases and phosphatases provides not only a new understanding of SMN function, but also constitutes a novel strategy for combinatorial therapeutic approaches to increase the level of SMN in SMA.

## 1. Introduction

Spinal Muscular Atrophy (SMA) is a neuromuscular disease characterized by loss of motoneurons in the brain stem and spinal cord and caused by deletion or mutation of the survival of motoneuron 1 (*SMN1*) gene [1]. The *SMN2* gene is highly similar but contains mutations leading to exclusion of exon 7 during pre-mRNA splicing [2]. The resulting truncated protein is unstable and cannot rescue the loss of *SMN1* [2,3]. As a consequence, muscle denervation at motor endplates is increased, leading to progressive muscle denervation, weakness, and atrophy [4]. The most severe form is SMA type I with an early onset before 6 months and a life expectancy smaller than two years. Copy number variation of *SMN2* can increase the amount of functional SMN protein [5] and leads to milder forms of the disease (SMA types II, III, and IV). Therapies have been approved very recently that include splicing correction of *SMN2* by an antisense-based approach and gene replacement therapy by systemic administration of an adeno-associated virus (AAV9) system coding for full-length SMN [6,7,8]. Although the results of these therapies are very impressive, there is a need for combinatorial approaches to further improve clinical outcomes. Moreover, the molecular pathomechanisms of SMA are still elusive.

The SMN protein is involved in a broad spectrum of molecular interactions [9]. The best-characterized function is its involvement in the biogenesis of small nuclear ribonucleoproteins (snRNPs). The so-called SMN complex (comprising its core components SMN, Gemins2–8 and unrip) promotes the ring-shaped assembly of the seven Sm proteins together with a U-rich small nuclear RNA (snRNA) [10,11,12,13]. After the cytoplasmic assembly of the pre-snRNP, the SMN complex guides it to distinct nuclear structures (Cajal bodies, CBs, or gems) for their maturation and release of mature tri-snRNPs [14]. Moreover, the redistribution of SMN from the cytoplasm into nuclear bodies is mediated by interaction of SMN with zinc finger protein ZPR1 in serum-induced response [15]. This ZPR1-driven translocation of SMN is disordered in SMA type I patients [15]. SMN is involved in a number of other cellular functions such as regulation of the neuronal actin cytoskeleton, signaling, and DNA repair [16,17,18,19]. However, less is known about the role of post-translational regulation on cellular functions of SMN.

Post-translational modifications such as phosphorylation regulate the function of SMN in a compartment-specific manner [20,21,22,23,24,25]. However, only little is known about the specific kinases and phosphatases acting on SMN and modulating its functional properties. For example, phosphorylation by protein kinase A (PKA) accelerates SMN accumulation in SMN-complexes as well as increases its binding to Gemin2 and Gemin8 and its stability [20,26,27], whereas dephosphorylation by the nuclear phosphatase PPM1G/PP2Cγ leads to accumulation of the SMN complex in CBs [21]. Importantly, several phosphorylation sites at the N-terminus regulate SMN protein stability, self-oligomerization, and the cytosolic assembly of the SMN complex [24]. Nonetheless, to the best of our knowledge, there are no studies showing functionality of the putative C-terminal phosphorylation sites as the C-terminal sequence renders SMN inaccessible for proteinases typically used in mass spectrometry (MS) analysis.

Amongst other cellular phenotypes, SMA motoneurons show a reduction of SMN-positive nuclear bodies (NBs). Thus, the understanding of post-translational modifications of SMN is also important for the development of combinatorial SMA treatments. A therapeutic strategy that improves SMN stability could enhance the benefits of approaches increasing SMN expression. In this context, we aimed to determine C-terminal phosphorylation sites and study their physiological functions. Here, we describe two novel phosphorylation sites, namely SMN S290 and S292. We analyzed the role of S290 with respect to protein interaction, protein stability, number of SMN-positive NBs and motoneuron survival in a *Caenorhabditis elegans* (*C. elegans*) model. Additionally, we identified phosphatase and tensin homolog (PTEN) as a phosphatase binding to SMN, and show that this function is influenced by pS290. We furthermore found that *Pten* knockdown or inhibition is detrimental for SMN stability and the number of SMN-positive NBs. These results raise new insights into SMN’s post-translational modifications and the regulatory impact on functionality and stability.

## 2. Materials and Methods

### 2.1. Cell Culture and Transfection

Motoneuron-like NSC34 cells (murine neuroblastoma × spinal cord hybrid cell line [28]) were incubated at 37 °C in a humidified atmosphere with 5% CO_2_. NSC34 cells were cultivated in proliferation medium (DMEM with GlutaMAX-I supplemented with 5% FBS, 100 U mL^−1^ penicillin and 0.1 mg/mL streptomycin). 18 h after seeding, medium was changed to low serum conditions (1% FBS). Cells were then transfected with plasmids or siRNAs using Lipofectamine2000 according to the manufacturer’s instructions and allowed to differentiate for three days. For analyzing oligomerization of the hSMN mutants, NSC34 cells were transfected with plasmids as mentioned above and then allowed to express transiently either the empty vector pEGFP-N2 or the different hSMN constructs for 48 h. Human embryonic kidney (HEK) 293T cells were cultivated in DMEM supplemented with 5% FBS, 100 U mL^−1^ penicillin and 0.1 mg/mL streptomycin and maintained at 37 °C in a humidified atmosphere with 5% CO_2_. To obtain high transfection rates for SMN-ZPR1 interaction assay, we used HEK cells that were transfected as mentioned above and either lysed or fixed 24 h post-transfection.

### 2.2. Plasmids, siRNAs and Inhibitors

The following siRNAs (Eurofins Genomics, Ebersberg, Germany) against murine *Pten* or *Smn* together with a control siRNA were used: siCtrl (GCGCAAAUAAACCGAAAGACA) [29], si*Pten* (CCAGAGGCUAGCAGUUCAA) and si*Smn* (CAGAAGUAAAGCACACAGCAA) [29].

Constructs coding for human full length (hSMN1-294), deleted (hSMN1-239, hSMN235-294) or mutant (hSMNY272C, hSMNT274I) SMN cloned into pEGFP-N2 were described previously [30,31]. hSMN HH227,273RR introducing an additional trypsin cleavage site for mass spectroscopy analysis was produced by site-directed mutagenesis (see below) in the same vector. SMNΔ7 cDNA was derived from human fibroblasts [31], PCR amplified using the following primers Fwd: CGGAATTCATGATGGCGATGAGCAGCGGCGGCAGTG and Rev: GCGTCGACTTGCCAGCATTTCCATATAATAGCC and cloned into pEGFP-N2 via EcoRI and SalI restriction sites. All inserts were fully sequenced. Plasmid constructs coding for the mutants pSMNS290A-EGFP and pSMNS290D-EGFP were synthesized by site directed mutagenesis of the plasmid coding for full-length SMN pSMN1-294-EGFP. Forward primers PC456-S290D (CAAAAAGAAGGAAGGTGCGACCATTCCTTAAA) and PC457-S290A (CAAAAAGAAGGAAGGTGCGCACATTCCTTAAA) were designed containing the amino acid exchange for the specific position. Reverse primer PC458-S290-REV (GCACCTTCCTTCTTTTTGATTTTGTCTGAAACCC) did not contain a mutation. For site-directed mutagenesis, the Phusion Site-Directed Mutagenesis Kit (Finnzymes/Thermo Fisher Scientific, Schwerte, Germany) has been used according to the manufacturer’s protocol.

The PTEN inhibitor bpV(HOpic) (Enzo ALX-270-206-M005, LOT08071510) was dissolved in DMSO added at a final concentration of 1 µM for indicated times. The proteasome inhibitor MG132 (Sigma, Darmstadt, Germany M7449-200UL, LOT 114M4018V) in DMSO was added at 10 µM for indicated times.

### 2.3. Immunoprecipitation

Cells were washed with PBS and lysed in IP buffer (25 mM Tris-HCl pH 7.5, 137 mM NaCl, 1% (*v*/*v*) IGEPAL, 10% (*v*/*v*) glycerol) for 30 min on ice. The lysate was passed 5 times through a 21 gauge needle and clarified by centrifugation at 4 °C at 15,000 rpm for 20 min. About 200 µg of protein were incubated with 1 µg of rabbit α-p-PTEN (S380/T382/T383) (Santa Cruz sc-101789, LOT D0113) or recombinant rIgG, respectively, overnight at 4 °C under gentle rotation. 15 µL of Dynabeads protein A or G (Invitrogen/Thermo Fisher Scientific, Schwerte, Germany) were added for 1 h at 4 °C rotating and beads were washed once with IP buffer and twice with PBS. Samples were boiled in Laemmli buffer for 5 min and used directly for SDS-PAGE.

To determine the SMN-ZPR1 interaction and the oligomerization state of the hSMN mutants, cell lysates were obtained as performed for Western blot analysis. Regarding oligomerization assay of the hSMN mutants, 200 µg of protein were incubated with 1 µg of mouse α-GFP (Roche, Mannheim, Germany, 11814460001, LOT 14442000) or 1 µg mIgG (Sigma) overnight at 4 °C under gentle rotation. For analyzing ZPR1 interaction in HEK 293T cells, 250 µg or 300 µg of total protein lysate were incubated with 1 µg of mouse α-ZPR1 (Invitrogen MA1-13003, LOT TG273632) or 1 µg mIgG (Sigma) overnight at 4 °C under gentle rotation. 15 µL Dynabeads protein G (Invitrogen) were added and incubated for 1 h at room temperature. Beads were washed twice with RIPA buffer and twice with PBS. Elution was performed with Laemmli buffer followed by boiling for 5 min. 5–10% of applied protein lysate served as input for Western blot analysis. Samples were stored at 4 °C till further processing for SDS-PAGE.

### 2.4. Mass Spectrometry

NSC34 cells were transfected with pEGFP-hSMN-HH227, 273RR and differentiated for 72 h. Upon lysis, as described above, SMN-EGFP was pulled down with a mouse α-GFP antibody (Roche 11814460001, LOT 14442000) bound to Dynabeads protein G, washed three times with IP buffer and eluted in Laemmli buffer. Proteins were separated by SDS-PAGE and the gel was stained with Coomassie Brilliant Blue (BioRad #161-0436). The expected band in the range of 60–65 kDa was excised and subjected to tryptic in-gel digestion as described previously [32]. To identify the protein composition of the gel band, 10% of the sample were desalted and analyzed by LC-MSMS. For sensitive detection of phospho-peptides, 90% of the extracted tryptic peptides from the gel band were enriched with the High-Select™ TiO_2_ Phosphopeptide Enrichment Kit (Thermo Fisher Scientific) following the manufacturer’s instructions. LC-MSMS analysis of peptides was performed on an Ultimate 3000 RSLC nano system online coupled to an Orbitrap Q Excative Plus mass spectrometer (both Thermo Scientific, Bremen, Germany). The system comprised a 75 µm i.d. × 250 mm nano LC column (Acclaim PepMap C18, 2 μm; 100 Å; Thermo Fisher Scientific). Data were acquired in a data-dependent acquisition mode using a Top10 method with the following parameters: full MS spectra, 300–1800 *m*/*z*, resolution of 70.000, AGC target 1 × 10^6^, max IT 50 ms, MSMS spectra, 200-2000 *m*/*z*, resolution 17.500; AGC target 5 × 10^5^, max IT 120 ms, 1^+^ charge state excluded, isolation window of 1.6 *m*/*z*, normalized collision energy of 27%). For phospho-peptides measurements, parameters were changed to AGC target 1 × 10^6^ and max IT 120 ms (MS), AGC target 5 × 10^4^ and max IT 500 ms (MSMS). Data were analyzed with Mascot software version 2.6.1 (Matrix Science Ltd., London, UK) and databases SwissProt 2018_07 (*Mus musculus* 17,043 sequences), a contaminant database (247 sequences) and an in-house-database (395 sequences including the entry of hSMN-HH227,273RR-EGFP). The following parameters were selected: trypsin/P, three missed cleavages, variable modifications, carbamidomethylation (C), propionamide, oxidation (M), phosphorylation (S, T, Y), Acetyl (Protein N-term), pyro-glu (Q), mass tolerances for MS and MSMS: 5 ppm and 0.02 Da. Proteins were accepted as identified if at least two unique peptides provided a Mascot MSMS score for identity (*p* < 0.01). In addition, MSMS spectra of phospho-peptides were validated by manual inspection.

### 2.5. Isoelectric Focusing

Isoelectric focusing (IEF) was carried out with the ZOOM Kit (Invitrogen) following the manufacturer’s instructions. In brief, cells were lysed in ZOOM Solubilizer 1 buffer containing protease inhibitors (Roche), chilled on ice for 10 min and sonicated at 50% power for 5 rounds under cooling. The lysates were clarified by centrifugation at 4 °C at 15,000 rpm for 20 min. Strips (pH 4–7) were rehydrated at RT overnight in a total volume of 155 µL containing 14.4 µL of sample, 138 µL ZOOM Solubilizer 1, 1.6 µL 1 M DTT, 1 µL carrier ampholytes and some bromophenol blue. IEF was carried out discontinuously: 200 V for 20 min, 450 V for 15 min, 750 V for 15 min and 2000 V for 105 min. The strips were incubated in 2× Laemmli buffer (125 mM Tris-HCl pH 6.8, 140 mM SDS, 20 mM DTT, 20% glycerol (*v*/*v*) and 0.03 g bromophenol blue) and directly used for SDS-PAGE. Shrimp alkaline phosphatase (SAP) control lysates were treated with 1 U SAP for 30 min at 37 °C prior to IEF.

### 2.6. Western Blot Analysis

Cells were washed with PBS, scraped into RIPA buffer containing protease and phosphatase inhibitors (Roche) and allowed to chill on ice for 20 min [33]. Afterward, lysates were sonicated and clarified by centrifugation (15,000 rpm, 20 min, 4 °C). Protein concentration was determined with the BCA Assay (Thermo Scientific). Equal amounts of protein were dissolved in Laemmli buffer, boiled for 5 min and separated by 12.5% SDS-PAGE. Protein was blotted onto a nitrocellulose membrane for 1 h at 120 V. Primary antibodies were: rabbit α-PTEN (Cell Signaling #9552S, LOT 0003, 1:1000), mouse α-SMN (BD Biosciences, Heidelberg, Germany, 610647, LOT 4157975, 1:4000), mouse α-α-tubulin (Santa Cruz sc-32293, LOT D0814, 1:4000), mouse α-GAPDH (Millipore, Darmstadt, Germany, MAB374, LOT 2910381, 1:4000), mouse α-GFP (Roche 11814460001, LOT 14442000, 1:4000), rabbit α-GFP (abcam ab290, LOT841067) and mouse α-ubiquitin (P4D1, Santa Cruz sc-8017, LOT B0817, 1:400). HRP-conjugated secondary antibodies (GE Healthcare, 1:4000) were used for visualization by chemiluminescence imaging using the Immobilon reagent (Millipore) or the SuperSignal^®^ West Femto Substrate (Thermo Scientific). Densitometric quantification was performed with the LabImage 1D software (Kapelan, Leipzig, Germany). Relative band intensities were calculated from bands of the same blot. Other targets were analyzed on the same blot after stripping of the membrane (62 mM Tris-HCl pH 6.8, 2% SDS and 0.7% β-ME) for 30 min at 50 °C.

### 2.7. Pulse-Chase Assay

On day in vitro 1 (DIV1) 1 × 10^6^ NSC34 cells were seeded in 10 mL proliferation medium per 100 × 20 mm cell culture dish and cultivated in a humidified atmosphere at 37 °C and 5% CO_2_. On DIV2, medium was changed to differentiation medium with low serum conditions (1% FBS). Cells were co-transfected with a siRNA against endogenous murine *Smn* and plasmid constructs coding for full-length SMN (pSMN1–294-EGFP) and the mutants pSMNS290A-EGFP or pSMNS290D-EGFP, respectively, as described above. Differentiation medium was changed after 6 h. On DIV3, cells were starved for 30 min in 4 mL methionine and cysteine depleted differentiation medium (DMEM high glucose without glutamine, methionine, and cysteine (Gibco™, Thermo Fisher Scientific, Schwerte, Germany, 21013024), 1% dialyzed FBS, 100 U mL^−1^ penicillin and 0.1 mg/mL streptomycin, 2 mM L-glutamine) followed by a radioactive pulse for 1 h: 100 µCi of [S35]-labeled methionine and cysteine mixture (Hartmann Analytic, Braunschweig, Germany, SRIS-103, 10 mCi/mL) were added to each dish. The radioactive pulse was stopped with 6 mL of ice-cold stop medium (differentiation medium supplemented with 100 µg/mL cysteine and 60 µg/mL methionine). Dishes were kept on ice for the subsequent steps. Medium was discarded and cells were washed twice with 5 mL ice-cold PBS. The chase was started with 10 mL pre-warmed chase medium (differentiation medium supplemented with 100 µg/mL cysteine and 60 µg/mL methionine) and incubated in a humidified atmosphere except for the first (0 h) time point. Cell samples were collected at different time points. The following steps were equal for all samples and time points. Chase medium was discarded, and cells were washed twice with ice-cold PBS followed by cell lysis with 300 µL RIPA (see above) buffer per dish. Cells were scratched off and dishes were washed with an additional 200 µL RIPA buffer. 9 µL DNaseI was added to each sample and homogenization was performed with a 27-gauge needle by pulling up and down ten times. Samples were centrifuged (20 min, 13,000 rcf, 4 °C) and supernatants were snap-frozen with liquid nitrogen and stored at −80 °C until further processing. Immunoprecipitation was subsequently performed at 4 °C. 400 µL of each sample was mixed with 2 µg of mouse α-GFP antibody (Roche 11814460001, LOT 14442000) or mIgG (Roche) as a control. After incubation overnight, 270 µg magnetic Dynabeads™ Protein G (Invitrogen) were added and samples were rotated for 1 h. The supernatant was separated using magnetic racks and frozen at −80 °C. Beads were washed twice with RIPA buffer and three times with PBS before resuspension in 60 µL Laemmli buffer and boiling at 95 °C for 3 min. Beads were pelleted (13,000 rcf, 1 min) and supernatant was stored at 4 °C until analysis by SDS-PAGE. After electrophoretic separation, gels were fixed in 10% (*v*/*v*) acetic acid, 25% (*v*/*v*) isopropanol for 30 min by shaking at room temperature. After fixation, gels were incubated for 30 min with scintillation solution (NAMP100, GE Healthcare) at room temperature. Gels were dried at 70 °C for 3 h and exposed to ECL films (Hyperfilm ECL, Thermo Fisher) backed by intensifying screens (Sigma Lite Plus, 104102LP) at −80 °C. Autoradiographs were digitalized and analyzed using ImageJ (Wayne Rasband; Version 1.41o).

### 2.8. C. elegans Experiments

Nematodes were grown and handled following standard procedures in uncrowded conditions at 20 °C on nematode growth medium (NGM) agar-plates seeded with *Escherichia coli* strain OP50 [34]. The transgenic strain used as recipient of injections was: NA1330 *gbIs4* [GBF109 *punc-25::smn-1(RNAi)*; GB301 *pchs-2::GFP*] III [35]. hSMN constructs (GB339 *punc-119*::hSMN WT; GB343 *punc-119*::hSMN S290A; GB344 *punc-119*::hSMN S290D) for pan-neuronal expression of hSMN WT, S290A and S290D were created as described above. cDNAs coding for full-length human SMN and the mutants SMNS290A and SMNS290D were cloned in to the *C. elegans* expression vector pBY103, a kind gift from O. Hobert (Columbia University, New York, NY, USA) that comprises the promoter *punc-119* [36]. Molecular cloning was performed by restriction digestions of plasmids with enzymes EcoRI and SalI. Germline transformation was accomplished as described by Mello et al., (1991) [37] by injecting into the gonad of NA1330 adult animals a DNA mixture containing the transgenic constructs above (20 ng/µL), together with pJM371 (*pelt-2::RFP*, RFP expression in the nuclei of intestinal cells) at 30 ng/µL, as a phenotypic co-injection marker to select the transgenic progeny. pJM371 [38] was a kind gift from Prof. J.D. McGhee (University of Calgary). At least two independent lines were examined for each transgenic strain: NA2112 *gbIs4* III; *gbEx659a/b* [GB339 *punc-119::hSMN WT; pelt-2::RFP*]; NA2163 *gbIs4* III; *gbEx665a/b/c* [GB343 *punc-119::hSMN S290A; pelt-2::RFP*]; NA2164 *gbIs4* III; *gbEx666 a/b/c* [GB344 *punc-119::hSMN S290D; pelt-2::RFP*].

### 2.9. Quantitative Reverse-Transcription PCR

Total RNA was isolated with the Qiagen RNeasy Kit following the manufacturer’s recommendations. 1 µg of RNA was mixed with 0.5 µL of Random Hexamers (Invitrogen) in a total volume of 12 µL and incubated at 70 °C followed by rapid cooling. Reverse transcription was carried out in a total volume of 40 µL containing 5× FS buffer (Invitrogen), 200 U M-MLV-transcriptase (Invitrogen), 40 U RNase-inhibitor (Agilent, Waldbronn/Ratingen, Germany), 0.02 µmol dNTPs (Invitrogen) and 0.4 µmol DTT (Invitrogen) at 42 °C for 90 min. The enzyme was heat-inactivated for 15 min at 70 °C. For qRT-PCR 5 µL of diluted cDNA (1:100), 7 µL of Power SYBR Green reaction mix (Applied Biosystems/Thermo Fisher Scientific, Schwerte, Germany) and 2 µL of primers (1.75 µM each) were mixed in a 96-well MicroAmp reaction plate (Applied Biosystems). qRT-PCR was performed using the StepOnePlus-thermocycler (Applied Biosystems) with the following program: 10 min 95 °C for initiation, 40 amplification cycles (15 s 95 °C and 60 s 60 °C) and recording of melting curves. Primer specificity was verified by melt curve analysis and agarose gel electrophoresis. Primers used for qRT-PCR (Eurofins MWG Operon) against murine *Smn* were *Smn*-Fwd (CCCTGGTCGACAAGAACAGAC) and *Smn*-Rev (ACGCTCTGCTGCTGACTTAGG) (Accession number NM_011420.2). As known to be stably expressed from earlier experiments, *Hprt1* was used as reference gene [39]. C_T_ values were determined with the StepOne-software V2.1 using a cycle threshold of 0.2. C_T_ values of technical duplicates had to be smaller equal 0.4 cycles and quantification was performed using the 2^−ΔCT^ method [40].

### 2.10. Immunocytochemistry

NSC34 cells or HEK 293T cells were washed with PBS and fixed with 4% PFA for 10 min or 5 min, respectively. Afterwards, cells were permeabilized with 1% (*v*/*v*) Triton X-100 and 4% (*v*/*v*) normal goat serum (NGS) in PBS for 20 min at RT. For analyzing ZPR1 and SMN co-localization, HEK 293T cells were permeabilized for 7 min and additionally incubated with ice-cold methanol for 5 min at −20 °C. Primary antibodies in PBS containing 1% (*v*/*v*) NGS were added for 1 h at room temperature or overnight at 4 °C. Primary antibodies were: rabbit α-PTEN (Cell Signaling #9552S, LOT 0003, 1:500), mouse α-ZPR1 (Invitrogen, MA1-13003, LOT TG273632, 1:200), and mouse α-SMN (BD Bioscience 610647, LOT 4157975, 1:1000). Alexa-coupled secondary antibodies (Invitrogen, 1:500) in PBS containing 1% horse serum were added for 1 h at RT. DAPI in PBS was added for 5 min at room temperature. Cells were mounted in Prolong Gold (Life Technologies).

### 2.11. Microscopy and Evaluation of Images

Confocal images were taken using a Leica TCS SP2 equipped with an oil immersion objective HCX PL APO BL (63×, numeric aperture 1.4) and with a ZEISS LSM 980 (40×, numeric aperture 1.4) with Leica acquisition software and ZEISS ZEN software (ZEN 2.3 SP1, ZEN 2.6 lite). Maximum intensity projection was performed for SMN-ZPR1 interaction studies. Background reduction, brightness, and contrast adjustment were performed over the whole image and kept equal for all images with the ImageJ software (ImageJ Image Processing and Analysis in Java. Available online: https://imagej.nih.gov/ij/ (accessed on 29.10.2020)). Co-localization was analyzed within the indicated ROIs using the Intensity Correlation Analysis plugin for ImageJ with default parameters after global background reduction (rolling ball method, radius 50 pixels) to determine product of the differences of the mean (PDM) values.

Epifluorescence images were taken with an Olympus BX60 upright fluorescence microscope with an Olympus XM10 Color View camera and Olympus Cell Sense software. For scoring dying motoneurons in *C. elegans*, animals were immobilized in 0.01% tetramisole hydrochloride (Sigma-Aldrich) on 4% (*w*/*v*) agar pads and visualized using a Zeiss Axioskop microscope equipped with epi-fluorescence. After *smn-1* silencing, dying motoneurons acquire fluorescence as a late sign of apoptosis and become visible without any motoneuron-specific expression of GFP [35]. For counting dying motoneurons, we scored the number of fluorescent motoneurons per animal. Epifluorescence images were collected with a Leica TCS SP8 AOBS microscope, using 63× objectives.

### 2.12. Quantification of Nuclear Bodies

Ten randomly picked images per coverslip were taken for quantification of SMN-positive nuclear bodies (CBs and nuclear gems). Frequency distributions were calculated for classes of cells with defined numbers of nuclear bodies. Values from technical replicates were averaged and treated as one biological replicate.

### 2.13. Statistics and Curve Fitting

The Statistical analyses were carried out using the GraphPad Prism 6 and 8 software (La Jolla, CA, USA). Numbers of biological replicates and tests performed are stated in the figure legends. Results were considered as significant if *p* < 0.05. Curve fitting of pulse-chase data was performed using an one-phase decay model.

## 3. Results

### 3.1. Identification of Novel Phosphorylation Sites in Human SMN

**Figure 1:**
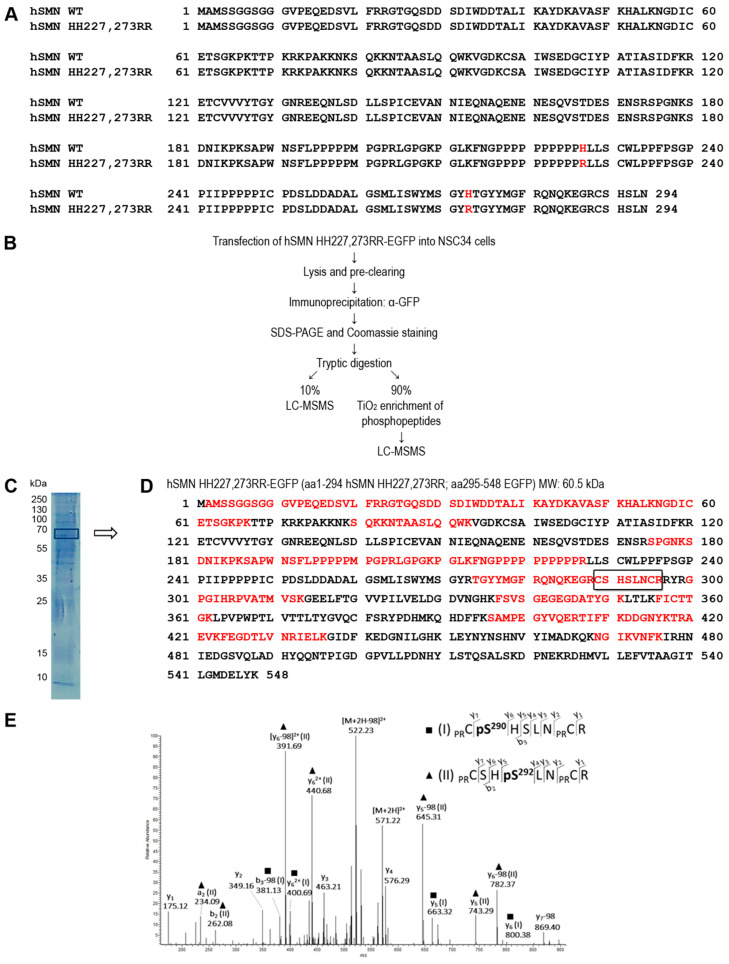
Phospho-analysis by mass spectrometry identifies human survival of motoneuron (hSMN) S290 and S292 as phosphorylation sites. (**A**) Sequence alignment of hSMN wildtype (WT) and mutant hSMN HH227, 273RR for trypsin cleavage at the C-terminus with the mutated site for additional trypsin cleavage sites shown in red. (**B**) The experimental workflow depicts the detection of phosphorylation sites in SMN. (**C**) SDS-PAGE and Coomassie staining of immunoprecipitated proteins. The box indicates the excised gel slice for MS analysis of SMN-EGFP. (**D**) LC-MSMS analysis after tryptic digestion identified hSMN HH227, 273RR-EFGP (10% sample). Framed sequence: the detected non-phosphorylated peptide ^289^CSHSLNCR^296^ comprising the C-terminus of hSMN HH227, 273RR (289–294) and two amino acid residues of the EGFP tag (295–296). (**E**) ESI-MSMS identified of the co-eluting phospho-peptides^289^_PR_CpS^290^HSLN_PR_CR^296^ and ^289^_PR_CSHpS^292^LN_PR_CR^296^ after enrichment with TiO_2_ (isobaric precursor ions *m*/*z* 571.218; charge 2^+^; _PR_C, propionamide of cysteine). Significant fragment ions for each peptide are indicated.

Full-length human SMN comprises 294 amino acid residues. Here, we aimed to analyze phosphorylation of the C-terminus. Tryptic digestion of SMN resulted in long peptides, which could not be analyzed by mass spectrometry (MS). Therefore, we employed a strategy that would increase MS accessibility to the C-terminal region of human SMN (hSMN), whereby we introduced two trypsin cleavage sites into a hSMN-EGFP construct through site-directed mutagenesis (denoted as HH227, 273RR; Figure 1A). The linker between SMN and EGFP introduced an additional arginine residue as a potential additional tryptic cleavage site. We then expressed this construct in differentiated motoneuron-like NSC34 cells and immuno-purified the mutated hSMN-EGFP protein with a GFP antibody bound to magnetic beads. After washing, we eluted the purified hSMN-EGFP fusion protein in reducing Laemmli buffer and analyzed its expression by SDS-PAGE and Coomassie staining followed by in-gel tryptic digestion (Figure 1B,C). To identify the protein composition of the gel band, 10% of the sample was analyzed directly by liquid chromatography MS/MS (LC-MS/MS) measurement. We detected hSMN-EGFP as the top protein with 27 unique peptides (*p* < 0.01) and a protein coverage of 43% (Figure 1D). Interestingly, the C-terminal peptide ^289^CSHSLNCR^296^ of hSMN was also identified (of which C295 and R296 are part of the linker sequence; Figure 1D). This peptide contains two putative phosphorylation sites within hSMN (S290 and S292) as predicted by NetPhos 3.1 analysis [41]. We were able to detect both sites (^289^CpS^290^HSLNCR^296^ and ^289^CSHpS^292^LNCR^296^) after phospho-peptide enrichment with TiO_2_ beads (Figure 1E). Together, we confirmed for the first time the putative phosphorylation residues hSMN S290 and S292 as novel SMN phosphorylation sites.

### 3.2. Characterization of hSMN S290 Phospho-Mutant and Phospho-Mimetic

**Figure 2:**
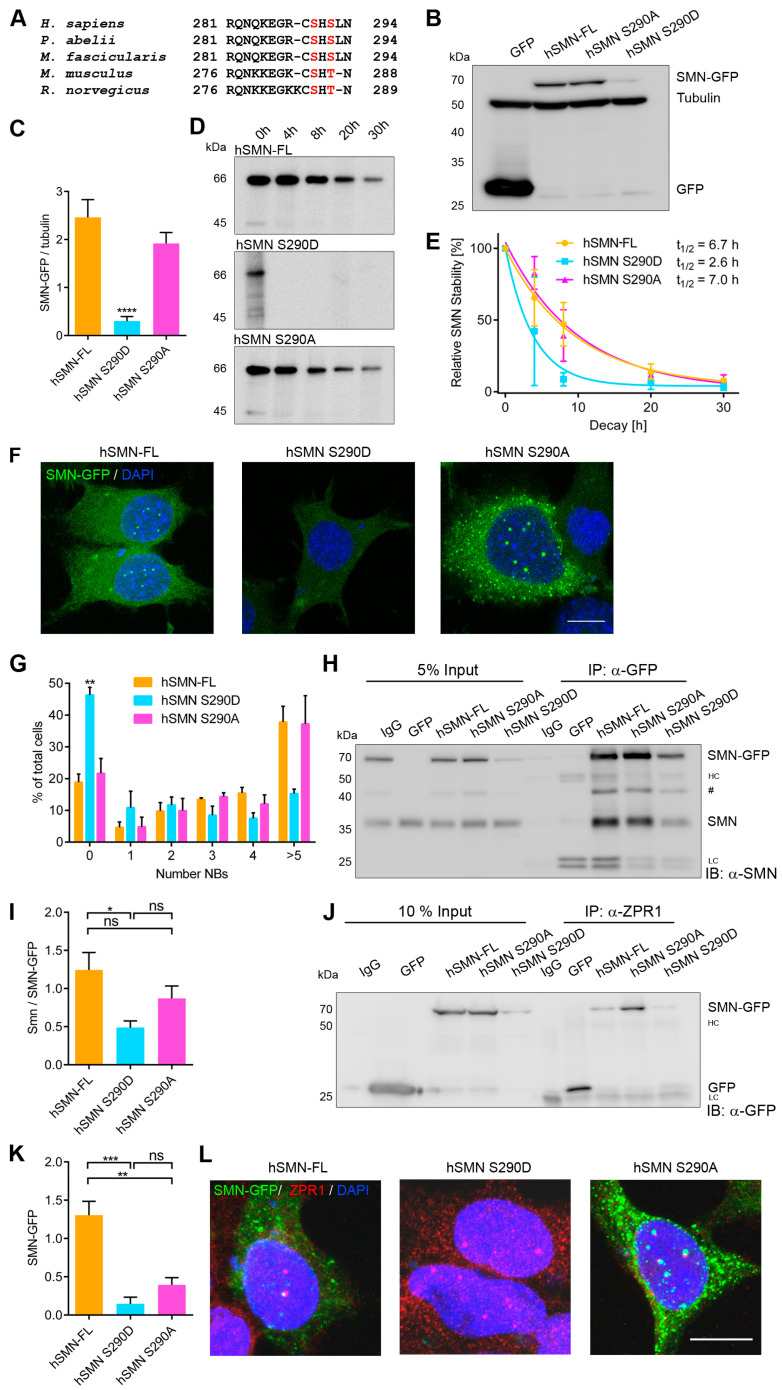
Characterization of hSMN S290 phospho-mutant and phospho-mimetic. (**A**) Multiple sequence alignment of the C-termini of SMN from different species showing conservation of serine 290 residue. (**B**,**C**) Expression of hSMN-FL, hSMN S290D and hSMN S290A in NSC34 cells by Western blot 48 h or 72 h post-transfection (mean ± SEM, *n* = 6, one-way ANOVA with Sidak post-test, **** *p* < 0.0001). (**D**,**E**) Radioactive pulse-chase experiment determined stability of hSMN-FL, hSMN S290D and hSMN S290A. Representative autoradiographs at the same exposure times and decay curves of immunoprecipitated hSMN-FL, hSMN S290D, and hSMN S290A (mean ± SEM, *n* = 3, non-linear regression, one phase decay, R^2^(hSMN-FL) = 0.815, R^2^(hSMN S290D) = 0.854, R^2^(hSMN S290A) = 0.846). (**F**) Representative fluorescence images display NSC34 cells expressing GFP-tagged hSMN-FL, hSMN S290D or S290A. Scale bar represents 20 µm. (**G**) Nuclear bodies (NBs) were quantified in NSC34 cells expressing hSMN-FL, hSMN S290D or hSMN S290A (mean ± SEM, *n* = 3, one-way ANOVA with Sidak post-test, ** *p* < 0.01). (**H**,**I**) NSC34 cells and (**J**,**K**) HEK 293T cells expressing GFP, GFP-tagged hSMN-FL, hSMN S290D and hSMN S290A (**H**,**I**) 48 h or (**J**,**K**) 24 h post-transfection. Co-immunoprecipitation of either (**H**,**I**) endogenous SMN and hSMN constructs or (**J**,**K**) ZPR1 and hSMN constructs were performed and determined by Western blot analysis. #, unspecific band. (**I**) Ratios of endogenous SMN to hSMN construct protein levels were quantified by densitometry and normalized to geometric mean (mean ± SEM, *n* = 4, unpaired *t*-test, * *p* < 0.05). (**J**) After immunoprecipitation (IP), we found a small amount of GFP in the empty vector control that is not visible in the other IP lanes. (**K**) Intensities of GFP-signals of hSMN constructs were quantified by densitometry and normalized to geometric mean (mean ± SEM, *n* = 4, unpaired *t*-test, * *p* < 0.05, ** *p* < 0.005). HC represents heavy chain, LC represents low chain. (**L**) Representative immunofluorescence of HEK 293T cells expressing GFP-tagged hSMN-FL, hSMN S290D or S290A (*n* = 3). Cells were additionally stained for ZPR1. Scale bar represents 10 µm.

The serine residue at the C-terminus of human SMN (hSMN S290) is conserved in humans, primates and rodents, whereas a serine at position 292 of hSMN is specific to humans and primates (Figure 2A). We therefore focused on hSMN S290 in the subsequent experiments. To characterize the physiological function of this amino acid residue, we constructed a non-phosphorylatable mutant (by replacing serine for alanine; S290A) and a phospho-mimetic (replacing serine by aspartic acid; S290D) of hSMN by site-directed mutagenesis. These constructs and wildtype (WT) hSMN-FL (full length) controls, respectively, were transfected into NSC34 cells and the expression was analyzed 48 h or 72 h post-transfection. Western blot analysis revealed that *hSMN-FL* and *hSMN S290A* were expressed at similar levels whereas *hSMN S290D* showed significantly decreased expression (Figure 2B,C). Since the measurements reflect steady states and we aimed to determine the stability of SMN and mutants quantitatively, we performed radioactive pulse-chase experiments (Figure 2D,E). Decay data were fitted using a one-phase decay model after normalization of t_0_ values. Half-life times revealed similar t_1/2_ values for hSMN-FL and mutant S290A, whereas S290D showed decrease of t_1/2_ to 39% compared to WT control. Therefore, the phospho-mimetic S290D displays an impaired stability, indicating a phosphorylation-dependent effect. In agreement with the expression analysis (Figure 2B), *hSMN S290D* also showed decreased expression at the zero hour time point compared to the control and *hSMN S290A* mutant (Figure 2D) on the autoradiographs.

To analyze the consequences of impaired stability mediated by phosphorylation of the single amino acid residue S290 in a cellular context, we next studied the number of SMN-positive nuclear bodies (NBs). Indeed, SMN localizes to these structures denoted as Cajal bodies and nuclear gems [42,43]. Cajal bodies are involved in modification and processing of snRNPs, a structure that becomes assembled by SMN in the cytoplasm before translocation to the nucleus. Phosphorylation at SMN’s N-terminus is important for proper assembly of the SMN complex and pre-snRNP formation of the splicing machinery [22,24,25]. Thus, low SMN levels decrease snRNPs and numbers of SMN-positive NBs, a phenotypic hallmark of SMA [44]. NSC34 cells were transfected with the constructs coding for GFP-tagged *hSMN-FL* and the *S290A* and *S290D* mutants. SMN-positive NBs were quantified, and frequency distributions were calculated (Figure 2F,G). S290D showed a significantly decreased number of SMN-positive NBs. This indicates impaired stability and/or incorporation in NBs of this phospho-mimetic compared to control and the non-phosphorylatable SMN.

In SMA patients with missense mutations within the C-terminal YG box of SMN, SMN fails to self-associate [45], and interaction of SMN with snRNPs is inhibited [46]. However, the mechanism of impaired oligomerization or failure in interaction by mutation of the C-terminus remains unclear. Here, we propose phosphorylation as a regulatory tool for self-oligomerization and protein interaction with ZPR1. Since SMN self-oligomerizes at the YG box and C-terminus [45,47] and zinc finger protein 1 (ZPR1) interacts with the SMN C-terminus recruiting cytoplasmic SMN into nuclear bodies [15,48], we next analyzed the impact of altered phosphorylation at S290 on these specific C-terminal SMN functions. Hence, immunoprecipitation served as a tool for determining the SMN oligomerization capacity and the interaction of SMN with ZPR1. The wildtype hSMN-FL as well as the mutants hSMN S290A and hSMN S290D all had the opportunity to bind equal amounts of endogenous SMN (Figure 2H). However, immunoprecipitation of wildtype hSMN and hSMN mutants by their GFP-tags revealed an impaired capacity of oligomerization of hSMN S290D with endogenous SMN compared to hSMN-FL (Figure 2H,I).

In contrast, hSMN S290A showed no difference in oligomerization with endogenous SMN compared to hSMN-FL or to its corresponding phospho-mimetic hSMN S290D. Taken together, these findings suggest that a phosphorylated state of SMN at S290 diminishes the oligomerization capacity of SMN. The expression of hSMN S290D was concomitantly reduced compared to hSMN-FL and hSMN S290A in input samples (Figure 2J) as shown earlier in NSC34 cells (Figure 2B,C). Additionally, hSMN-FL and hSMN S290A showed a similar input signal in contrast to the corresponding immunoprecipitation: hSMN S290A revealed an increased binding to ZPR1 as its stronger signal indicates compared to hSMN-FL signal (Figure 2J,K). Immunocytochemistry revealed co-localization of either hSMN-FL or hSMN-S290A with ZPR1 in nuclear bodies (Figure 2L). Regarding hSMN S290D, binding to ZPR1 was strongly impaired in contrast to hSMN-FL and to its non-phosphorylatable mutant S290A (Figure 2J,K) and failed in co-localization with ZPR1 (Figure 2L). Considering (i) the phosphorylation as a dynamic post-translational modification and (ii) the binding domain of ZPR1 at SMN C-terminus, the phosphorylation state at S290 may modulate the interaction of SMN with ZPR1. Nonetheless, the interaction of SMN and ZPR1 may not be exclusively restricted to a steady state of phosphorylation at S290.

**Figure 3:**
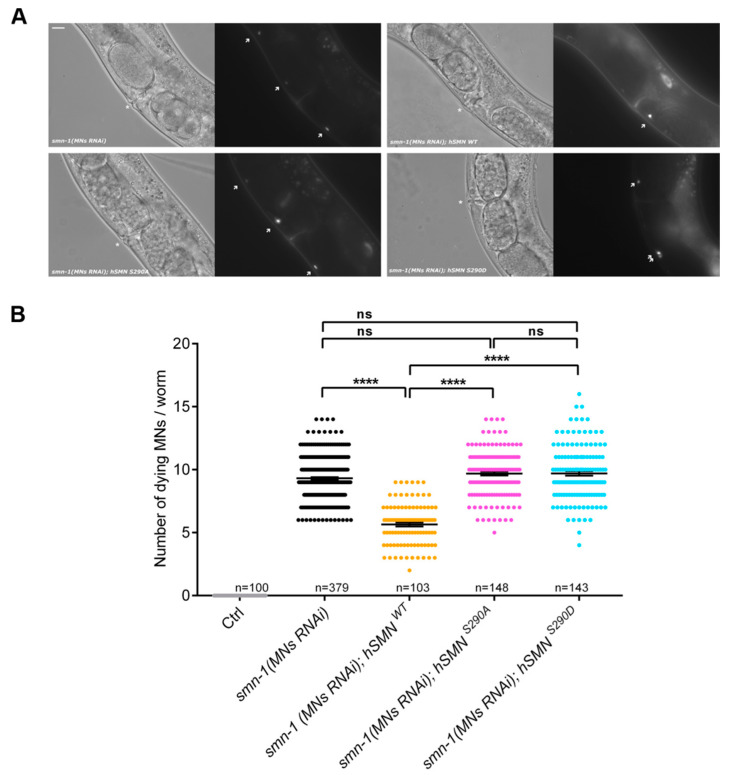
Phosphorylation state of serine residue 290 (S290) affects motoneuron survival in a *C. elegans* Spinal Muscular Atrophy (SMA) model**.** (**A**) Representative fluorescence images show dying MNs. *smn-1(MNs RNAi); hSMN WT* animals (upper right panel) presents less dying neurons (arrows) than *smn-1(MNs RNAi)* (upper left panel) and *smn-1(MNs RNAi); hSMN S290A* and *smn-1(MNs RNAi); hSMN S290D* animals (lower left and right panels, respectively). * indicates the vulva localized at mid-body. In all panels, anterior is left and ventral is down. Scale bar represents 10 µm. (**B**) Quantification of the effects of *hSMN WT, hSMN S290D,* and *hSMN S290D* on motoneuron survival in a *C. elegans* SMA model. Each dot represents the number of dying motor neurons in a single animal. The mean number of dying motoneurons and the SEM is also shown. The number of animals scored is annotated (n). One-way ANOVA with Kruskal-Wallis test, **** *p* < 0.0001, ns = not significant.

To explore the in vivo role of the hSMN S290 residue and its involvement in SMN function in motoneurons, we tested the effects of hSMN S290A and S290D on motoneuron survival in a *C. elegans* model of SMA (Figure 3A,B). *smn-1*, the ortholog of human *SMN1* in *C. elegans*, is ubiquitously expressed and its depletion causes larval arrest [49,50]. To avoid the pleiotropic effects of *smn-1* depletion, we used a transgenic model with silencing of *smn-1* specifically in D-type motoneurons [*smn-1*(*MNs RNAi*)]. These animals are viable and fertile but present impairment in locomotion and motoneuron death [35]. Thus, we generated and injected three constructs expressing *hSMN WT, hSMN S290A* and *hSMN S290D* under the control of a pan-neuronal promoter in a *smn-1*(*MNs RNAi*) background. The expression of *hSMN WT* partially rescued the neuronal death shown by *smn-1*(*MNs RNAi*), reducing the average number of dying motor neurons from 9.32 to 5.65 (Figure 3A,B).

We also observed that neither the non-phosphorylatable mutant (*hSMN S290A*) nor the phospho-mimetic (*hSMN S290D*) were able to rescue the neuronal death of *smn-1*(*MNs RNAi*), with 9.69 and 9.70 dying MNs, respectively (Figure 3A,B). These results demonstrate that the serine residue in position 290 is essential for SMN function in *C. elegans* motoneurons.

### 3.3. PTEN is a Novel SMN-Interacting Partner

Since we showed that phosphorylation of hSMN S290 is detrimental for its stability and physiological function, we next sought to identify a phosphatase that dephosphorylates this residue and stabilizes SMN. We recently found that PKB/AKT is hyperphosphorylated in Smn-depleted NSC34 cells [29]. The main upstream regulators of PKB/AKT are the class I phosphatidylinositol-3-kinases (PI3Ks) and phosphatase and tensin homolog (PTEN) [51]. Apart from its role as a tumor suppressor and antagonist of the PI3K/AKT pathway [52], PTEN is an important phosphatase during CNS development, regulating soma size, neurite branching and arborization, as well as axonal pathfinding, which involve its lipid and protein phosphatase activities [53]. *PTEN* mutations or even subtle protein level changes are frequently linked to macrocephaly, autism spectrum disorders, epilepsy and Cowden syndrome [54,55]. Interestingly, it has been described recently that PTEN binds to spliceosomal components including U2AF in HEK293T cells to regulate alternative splicing and that PTEN depletion resulted in alternative splicing events linked to tumorigenesis [56]. Additionally, the interaction between PTEN and U2AF as well as other spliceosomal components (e.g., SF3B2 or LSM5) was also confirmed in stressed neurons [57].

**Figure 4:**
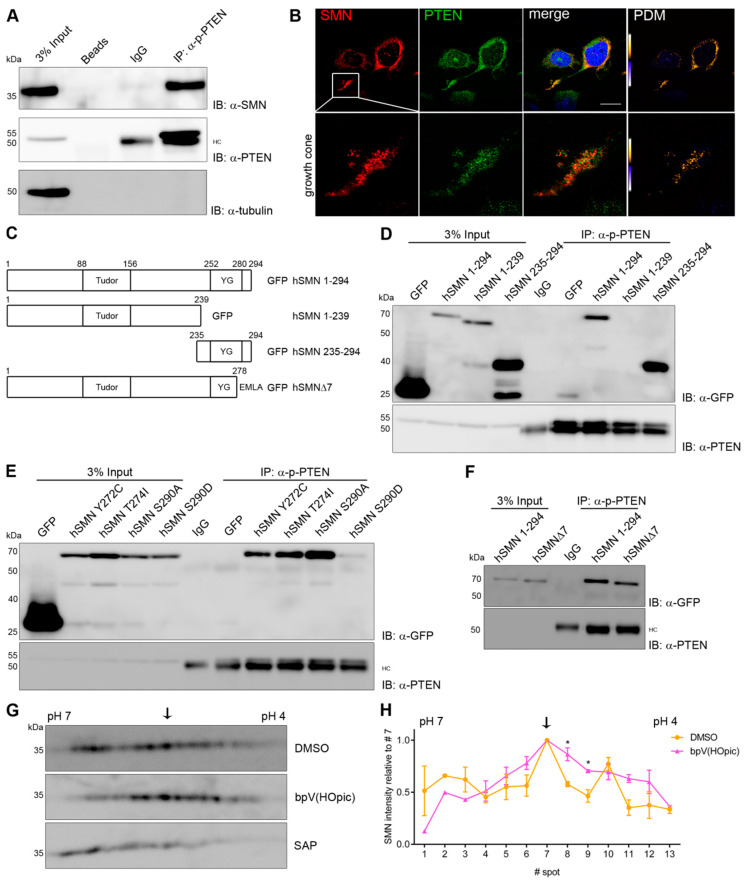
Endogenous phosphatase and tensin homolog (PTEN) binds to the C-terminus of SMN and dephosphorylates SMN. (**A**) Co-immunoprecipitation of endogenous p-PTEN and SMN from lysates of differentiated NSC34 cells. Beads alone, IgG and tubulin were used as negative controls. HC represents heavy chain. (**B**) Confocal images of NSC34 cells were stained for endogenous SMN and PTEN together with their respective PDM images. Scale bar represents 20 µm. (**C**) Schematic overview of hSMN constructs used in immunoprecipitation assays. Cells were transfected and prepared for the assay 18 h post-transfection. (**D**–**F**) Co-immunoprecipitation assays with hSMN deletion constructs (**D**), patient mutations (**E**) and SMNΔ7 (**F**) 18 h post-transfection. (**G**,**H**) Isoelectric focusing of SMN from NSC34 cells treated with either DMSO, PTEN inhibitor bpV(HOpic) or shrimp alkaline phosphatase (SAP) for 2 h prior to lysis. Spot #7 (indicated by arrow) was used as reference (mean ± SEM, *n* = 3, paired two-tailed *t*-test, * *p* < 0.05).

To analyze whether PTEN and SMN are endogenous interactors, we performed co-immunoprecipitations (IPs) with non-transfected and differentiated NSC34 cells (Figure 4A). After precipitation of phospho-PTEN (p-PTEN; S380/T382/T383), a strong signal of endogenous SMN could be detected by Western blot (IB; Figure 4A), indicating an endogenous interaction between PTEN and SMN. An immunoglobulin (IgG) control, beads alone as well as incubation of the membrane with an antibody for α-tubulin served as negative controls. None of them showed any signals, thus suggesting a specific interaction between p-PTEN and SMN. Of note, since a pan-PTEN antibody, directed to the C-terminus of PTEN, did not work in the immunoprecipitation assays, we employed a phospho-PTEN (p-PTEN) antibody in these assays. PTEN exists in two conformations: (i) the open non-phosphorylated form that preferentially binds to the plasma membrane to dephosphorylate PtdIns(3,4,5)P3 to PtdIns(4,5)P2, and (ii) a closed phosphorylated form preferentially localized in the cytosol and nucleus [58]. The IP results suggest that PTEN prefers to bind to SMN in the closed, phosphorylated conformation. To verify a physiological interaction between SMN and PTEN, we carried out immunocytochemistry experiments in NSC34 cells with antibodies against SMN and pan-PTEN (Figure 4B). Endogenous SMN and PTEN co-localized within the cytosol and in growth cones but not in the nucleus (e.g., in gems or CBs). A quantitative evaluation of the co-localization by calculation of product of the differences of the mean (PDM)-values [59] revealed positive values confirming the qualitative co-localization patterns (Figure 4B). The results demonstrate an interaction between PTEN and SMN in a common complex, which localized to growth cones and the cytosol.

We next addressed which domain of SMN is involved in PTEN binding. NSC34 cells were transfected with either of the following constructs fused to GFP: full-length hSMN 1–294, a C-terminal deletion mutant hSMN 1–239 (lacking residues 240–294) and an N-terminal deletion mutant hSMN 235–294 (Figure 4C) [30]. 18 h post-transfection, p-PTEN was pulled down and analyzed for co-precipitation of SMN using an antibody against GFP. hSMN 1–294 and N-terminal deleted hSMN 235–294 were co-immunoprecipitated whereas SMN, lacking its C-terminus (hSMN 1–239), was not detected (Figure 4D), indicating that PTEN binds to SMN at its C-terminus. We additionally tested whether SMNΔ7, which lacks the last 16 amino acid residues compared to hSMN 1–294 and is the main SMN isoform in SMA due to alternative splicing, harbors PTEN binding properties. Indeed, SMNΔ7 co-immunoprecipitated with PTEN (Figure 4F). These results suggest that the interaction of PTEN with the C-terminus of SMN comprises at least the residues 239–278.

As mentioned above, loss of *SMN1* is the main cause of SMA. Nevertheless, there are a few patients harboring at least one allele of a non-deleted *SMN1* that carries missense mutations [60]. We tested two known patient mutations within the C-terminus (hSMN Y272C and T274I) in co-immunoprecipitation assays with regard to PTEN binding (Figure 4E). The hSMN Y272C and hSMN T274I mutations, which localize to the highly conserved YG-box of SMN (corresponding to the hSMN 235–294 construct) and inhibit SMN self-oligomerization [46], did not alter the interaction with PTEN. Additionally, we tested the phospho-mutant and phospho-mimetics hSMN S290A/D in binding assays. We found that hSMN S290A was strongly bound to PTEN whereas hSMN S290D was hardly precipitated (Figure 4E). Taken together, these results show that PTEN binding to hSMN is not altered by the selected patient mutations Y272C and T274I but that mimicking phosphorylation of hSMN S290 influences PTEN-binding.

### 3.4. PTEN Dephosphorylates SMN In Vitro

We showed that PTEN binds the C-terminus of SMN and that specific patient mutations do not influence binding, whereas mimicking phosphorylation at hSMN S290 abolishes the interaction with

PTEN. Next, we wanted to evaluate whether PTEN not only binds to but also dephosphorylates SMN. To test this, NSC34 cells were treated with the PTEN inhibitor bpV(HOpic), a bisperoxovanadium (bpV) compound [61], or solvent for two hours. Subsequently, SMN protein was analyzed by 2D Western blot (1st dimension: isoelectric point determined by isoelectric focusing; 2nd dimension: apparent molecular weight separation by SDS-PAGE) (Figure 4G). Additional lysates treated with shrimp alkaline phosphatase (SAP) were included as controls to show that the SMN distribution pattern in the gel is caused by phosphorylation. If PTEN inhibition leads to decreased SMN dephosphorylation, an increase of acidic spots is expected due to negatively charged phospho-groups.

PTEN inhibition with bpV(HOpic) indeed led to a shift in SMN intensity from the neutral to the acidic side compared to control. The most prominent spot in both conditions (spot #7, arrows in Figure 4G,H) served as a reference spot for quantification (Figure 4H). In contrast, neutral SMN spots adjacent to the core spot became weaker in intensity. This shift upon PTEN inhibition therefore indicates that PTEN is a *bona fide* phosphatase for SMN.

### 3.5. Pten Knockdown Negatively Influences SMN Stability and Reduces the Number of SMN-Positive Nuclear Bodies

**Figure 5:**
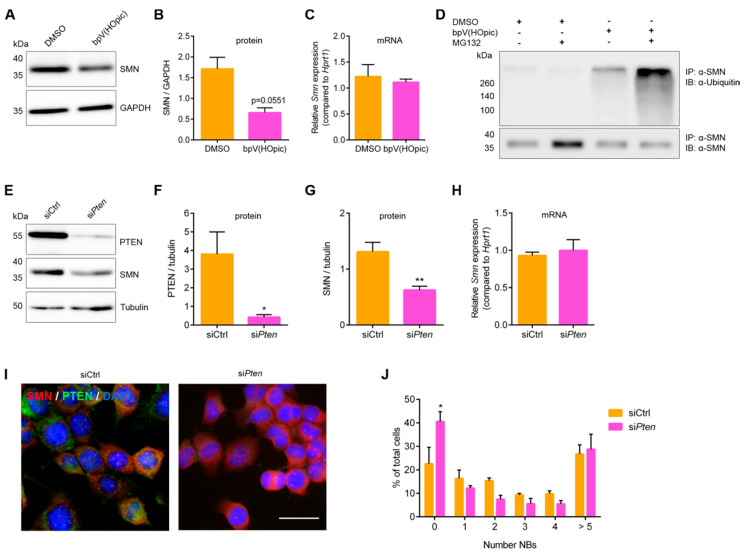
PTEN inhibition and knockdown decrease SMN levels via proteasomal degradation. (**A**,**B**) NSC34 cells were differentiated and treated with DMSO or PTEN inhibitor bpV(HOpic) for 6 h prior to lysis. Western blot analysis determined SMN protein levels and SMN compared to housekeeping protein was quantified by densitometry (mean ± SEM, *n* = 5, paired two-tailed *t*-test, *p* = 0.0551). (**C**) Relative *Smn* expression of DMSO- or bpV(HOpic)-treated cells (6 h) was analyzed by qRT-PCR (mean ± SEM, *n* = 3, paired two-tailed *t*-test, ns = not significant). (**D**) Cells were differentiated and treated with DMSO or bpV(HOpic) and in the absence or presence of the proteasome inhibitor MG132 for 6 h. SMN ubiquitination was analyzed upon SMN immunoprecipitation. (**E**–**G**) NSC34 cells were transfected with siRNA for *Pten* (si*Pten*) or control (siCtrl) and differentiated. Western blot analysis determined PTEN and SMN levels (**E**) together with densitometric quantification of PTEN (**F**) and SMN (**G**) compared to housekeeping protein (mean ± SEM, *n* = 5, paired two-tailed *t*-test, * *p* < 0.05, ** *p* < 0.01). (**H**) Relative *Smn* expression in control or PTEN-depleted cells analyzed by qRT-PCR (mean ± SEM, *n* = 3, paired two-tailed *t*-test, ns). (**I**,**J**) Quantification of SMN-positive nuclear bodies (NBs) of control or PTEN-depleted cells (mean ± SEM, *n* = 3, multiple *t*-test, * *p* < 0.05).

We next wondered about functional effects mediated by altered SMN phosphorylation. Since we have shown that (i) hSMN S290 regulates PTEN binding, (ii) PTEN dephosphorylates SMN, and (iii) hSMN S290D is unstable, we wanted to test the hypothesis that PTEN inhibition negatively influences SMN stability. To address this point, we first analyzed the endogenous SMN protein level after 6 h of PTEN inhibition with bpV(HOpic). Interestingly, the level of SMN protein but not of mRNA was markedly decreased (~60%, *p* = 0.0551) compared to control (Figure 5A–C). SMN has a typical half-life of about 6–24 h depending on the cell type [26,62,63,64] and is mainly degraded by the proteasome [26,65].

As we consistently observed a SMN half-life of 6–7 h (Figure 2E) and SMN reduction after 6 h of PTEN inhibition, we wondered whether phosphorylation primes SMN for ubiquitination and proteasomal degradation. To investigate this, cells were treated with DMSO or bpV(HOpic) and with or without the proteasome inhibitor MG132 for 6 h followed by immunoprecipitation of SMN and analysis of its poly-ubiquitination state by Western blot. We observed concomitant lowered SMN levels and increased SMN poly-ubiquitination upon PTEN inhibition (Figure 5D). Since the SMN reduction upon PTEN inhibition was not significant, we genetically knocked down *Pten* using an RNAi approach. Treatment with siRNA against murine *Pten* (si*Pten*) led to 85% PTEN protein level reduction compared to control (siCtrl) (Figure 5E,F). Concomitantly with *Pten* knockdown, SMN protein levels significantly decreased by about 50% (Figure 5G), whereas mRNA was unaffected (Figure 5H), similar to the PTEN pharmacological inhibition experiment. Taken together, we propose that PTEN is necessary to dephosphorylate and stabilize SMN, thus preventing its degradation.

Since we have shown in our model that SMN levels are reduced and that SMN phosphorylation is altered upon PTEN depletion or inhibition, we wondered whether *Pten* knockdown also influences SMN-positive NB numbers. As expected, we observed that *Pten* knockdown in NSC34 cells had a negative impact on the number of NBs (Figure 5I,J). The percentage of PTEN-depleted cells without NBs significantly increased by two-fold compared to control cells. Additionally, the percentage of cells with 1–4 NBs was consistently decreased compared to control cells. Our results indicate that PTEN is necessary for the presence of SMN-positive NBs.

## 4. Discussion

In this study, we have shown for the first time that phosphorylation of serine 290 is detrimental to the stability of SMN. Moreover, PTEN is a protein phosphatase dephosphorylating SMN and thereby stabilizing the protein. Interestingly, AAV-mediated *Pten* knockdown resulted in increased survival of motoneurons isolated from SMNΔ7 SMA mice [66]. In the same mice, ameliorated neuromuscular junction pathology, motor performance, and modestly prolonged lifespan were observed when *Pten* had been systemically knocked down [67]. Mechanistically, PTEN depletion increased the antiapoptotic activity of AKT as well as AKT’s downstream effector mTOR, which may be the primary neuroprotective effects following *Pten* knockdown [68]. Although we show that PTEN depletion is detrimental for SMN stability, such effects were not detected in these previous studies. However, (i) the effect of PTEN depletion on SMN levels had not been analyzed in WT, (ii) SMNΔ7 does not harbor residue S290, and (iii) we have analyzed SMN stability in a motoneuron-like cell line in contrast to muscle cells in other studies. The *C. elegans* PTEN homologue DAF-18 and PTEN share conserved sequences [69] and DAF-18 depletion increases motoneuron regeneration in *C. elegans* [70], corroborating published data regarding PTEN depletion. However, effects of DAF-18 loss in *smn-1* background of the *C. elegans* SMA model could be limited due to differences in sequence similiarity between SMN of *C. elegans* and human as our rescue experiment with hSMN WT has shown. Thus, further in vivo investigations are needed to elucidate the functional role of PTEN in SMA.

A small number of phosphatases have already been discovered that regulate SMN localization and function [21,23,25]. In this study, we identified PTEN as a novel endogenous interactor and phosphatase of SMN and show that PTEN is necessary for formation of SMN-positive NBs. Our results additionally suggest that PTEN regulates splicing complexes on another level that is by promoting the cytosolic SMN complex and snRNP precursor formation, since SMN protein stability and NB numbers decreased upon PTEN knockdown due to phosphorylation changes. Here, we found that hSMN residues S290 and S292 are phosphorylation sites and show that S290 plays a physiological function in vivo, a function that has been conserved in evolution. Our results show that PTEN binding is modulated by S290. However, this site is not an exclusive regulator since SMNΔ7 could partially bind PTEN. Based on our binding assays, in which we narrowed the PTEN binding site down to residues 239–278, it is also possible that hSMN S290 is the target site for PTEN.

S290 localizes to the sequence encoded by exon 7. Deletion of exons 6 or 7 decreased half-life of SMN by approximately half, indicating an important role of those sequences for regulation of SMN stability [26]. Moreover, this region is involved in self-association of SMN, direct binding of Gemin3, and indirect interaction with Gemin4 as well as the zinc finger protein ZPR1 [2,13,15,71]. Focusing on defined C-terminal functions of SMN, we found that SMN oligomerization was impaired in the phospho-mimetic hSMN S290D. Furthermore, hSMN S290D failed in binding to and co-localization with ZPR1. Of note, hSMN S290D shows several impairments in SMN functions, which are not prominent in hSMN S290A. However, this does not necessarily imply that the non-phosphorylated hSMN S290A shows completely adverse effects. Therefore, we suppose that regulation of SMN stability is not restricted to the phosphorylation state at S290 only.

A first antisense oligonucleotide (ASO)-based therapy (Nusinersen/Spinraza™) has been approved in 2016 by the FDA and in 2017 by European Medicines Agency (EMA). However, it is currently unclear to what extent possible non-motoneuron phenotypes might emerge in treated severely affected patients and whether all patients can be successfully treated with this approach since about 50% of the SMA type I patients are non-responders [6,7,72]. Treatment possibilities have recently been extended by approval of an AAV9-based gene replacement therapy onasemnogene abeparvovec-xioi (Zolgensma™) [8] and the systemically administered small compound Risdiplam (Evrysdi™). This changing therapeutic landscape has identified the need for novel combinatorial treatments that consist of SMN-independent and -dependent treatment strategies that can support or augment the benefits of the currently approved SMN gene therapies [73]. The identification of SMN phosphatases and kinases is of great importance for the development of such new strategies that could stabilize the SMN protein and maximize its functional benefits.

In summary, our data provide novel insights into post-translational modification of the SMN protein, which is of importance for putative future combinatorial SMA therapies comprising expressional increase and stabilization of SMN. Although many kinases display pleiotropic effects, novel approaches aim to target specifically the substrate-kinase interface [74]. Inhibition of the kinase responsible for phosphorylation of serine residues at the C-terminus could be such a putative strategy.
